# Esophageal Granular Cell Tumor: An Uncommon Cause of Dysphagia

**DOI:** 10.7759/cureus.41846

**Published:** 2023-07-13

**Authors:** Richard A Revia, Raj Shah, Amar Mandalia, Jignesh Parikh, Vania Zayat

**Affiliations:** 1 Pathology, University of Central Florida College of Medicine, Orlando, USA; 2 Internal Medicine, University of Central Florida Hospital Corporation of America (HCA) Healthcare Graduate Medical Education, Orlando, USA; 3 Internal Medicine, Orlando Veterans Affairs Medical Center, Orlando, USA; 4 Gastroenterology, Orlando Veterans Affairs Medical Center, Orlando, USA; 5 Pathology, Orlando Veterans Affairs Medical Center, Orlando, USA

**Keywords:** case report, histopathology, dysphagia, esophagus, granular cell tumor

## Abstract

Granular cell tumors (GCTs) are rare, typically benign, solitary neoplasms that can arise throughout the body, with reports of cases in the tongue, esophagus, colon, skin, vulva, and skeletal muscle, among others. Although GCTs are usually asymptomatic, esophageal GCTs can grow large enough to cause dysphagia. When developing the differential diagnosis for dysphagia, a broad consideration includes routine etiologies such as esophageal strictures, eosinophilic esophagitis, carcinoma, webs and rings, achalasia, and motility disorders, but GCTs may not readily come to mind. Due to their scarcity, this case report is presented to raise awareness of esophageal GCTs and emphasize key goals for diagnosing and managing this uncommon yet treatable cause of dysphagia. This case report details the clinical course of a patient presenting with a chief complaint of difficulty swallowing that was found to be caused by a subepithelial esophageal tumor discovered with esophagogastroduodenoscopy (EGD) and endoscopic ultrasound (EUS). Histopathological studies paired with immunohistochemical investigations of a tissue biopsy confirmed the etiology of the offending esophageal mass to be a GCT. The patient’s dysphagia resolved after endoscopic mucosal resection of the GCT, and follow-up evaluations have remained negative for recurrence. This case highlights the esophageal GCT as an uncommon source of dysphagia and the need for EGD and EUS evaluations of subepithelial esophageal lesions accompanied by histopathological analysis for a definitive diagnosis of GCT.

## Introduction

The origin of the name of the neoplasm known as a granular cell tumor (GCT) becomes evident when microscopic tissue studies of a GCT reveal ample coarse, eosinophilic granules populating the cytoplasms of disorganized sheets of tumor cells. The borders of GCTs are often irregular and may be so poorly defined as to suggest an invasive nature [1]. However, in most cases, GCTs are benign, with estimates of the proportion of malignant forms ranging from 2 to 4% [2-4]. Documented instances of GCTs most often describe solitary lesions, although sporadic occurrences of GCTs presenting multifocally are estimated to compose 5 to 7% of cases [1-3]. The largest portion of GCTs form in the gastrointestinal tract, followed by the skin as the second-most common site of origin and the respiratory tract as the third-most common site [2,3]. Gastrointestinal GCTs are most commonly found in the distal esophagus [2,3,5]. Despite the propensity for GCTs to form in the gastrointestinal tract, esophageal GCTs are rare entities representing approximately 1% of all esophageal lesions [6]. Moreover, retrospective case reviews have shown that esophageal GCTs are found in less than 0.02% of esophagogastroduodenoscopies (EGDs) and less than 0.1% of all esophageal biopsies [5,7].

Generally, GCTs are asymptomatic regardless of location. However, GCTs emerging near or within the esophagus can increase in size to form a bulge that blocks the esophageal lumen leading to dysphagia [8]. Here, we present a case report of an esophageal GCT to highlight this rare cause of dysphagia. This case exemplifies why GCT should be among the diagnoses when considering subepithelial esophageal masses.

## Case presentation

**Figure 1:**
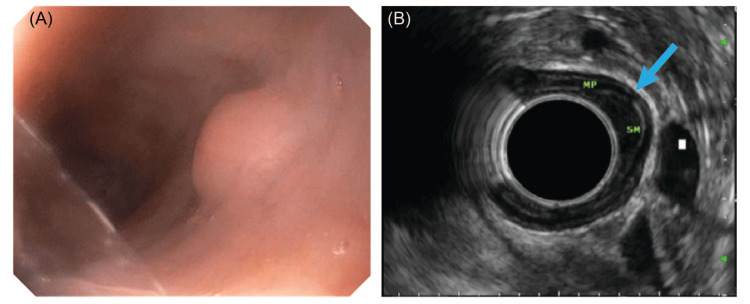
(A) EGD showing a raised submucosal nodule in the middle third of the esophagus with a smooth surface. (B) EUS reveals a submucosal oval 8 mm × 3 mm intramural hypoechoic lesion (marked by the blue arrow). EGD, esophagogastroduodenoscopy; EUS, endoscopic ultrasound

A 68-year-old male with a past medical history of gastroesophageal reflux disease, erosive esophagitis, ulcerative colitis, and diverticulosis presented to the clinic complaining of a 10-month history of intermittent dysphagia to both solids and liquids but an absence of odynophagia. Physical exam findings and routine laboratory values were unremarkable. Before further evaluation could be achieved with EGD, the patient failed to attend scheduled medical appointments and lost contact with his medical providers; however, he returned to the clinic two years later with persistent difficulty swallowing. At this time, EGD revealed a smooth, round sessile nodule measuring 12 mm along its greatest dimension that projected into the lumen of the middle third of the esophagus, as shown in Figure 1A. Subsequent evaluation with endoscopic ultrasound (EUS) showed a single hypoechoic, oval 8 mm × 3 mm intramural lesion originating within the submucosa, as shown in Figure 1B.

**Figure 2:**
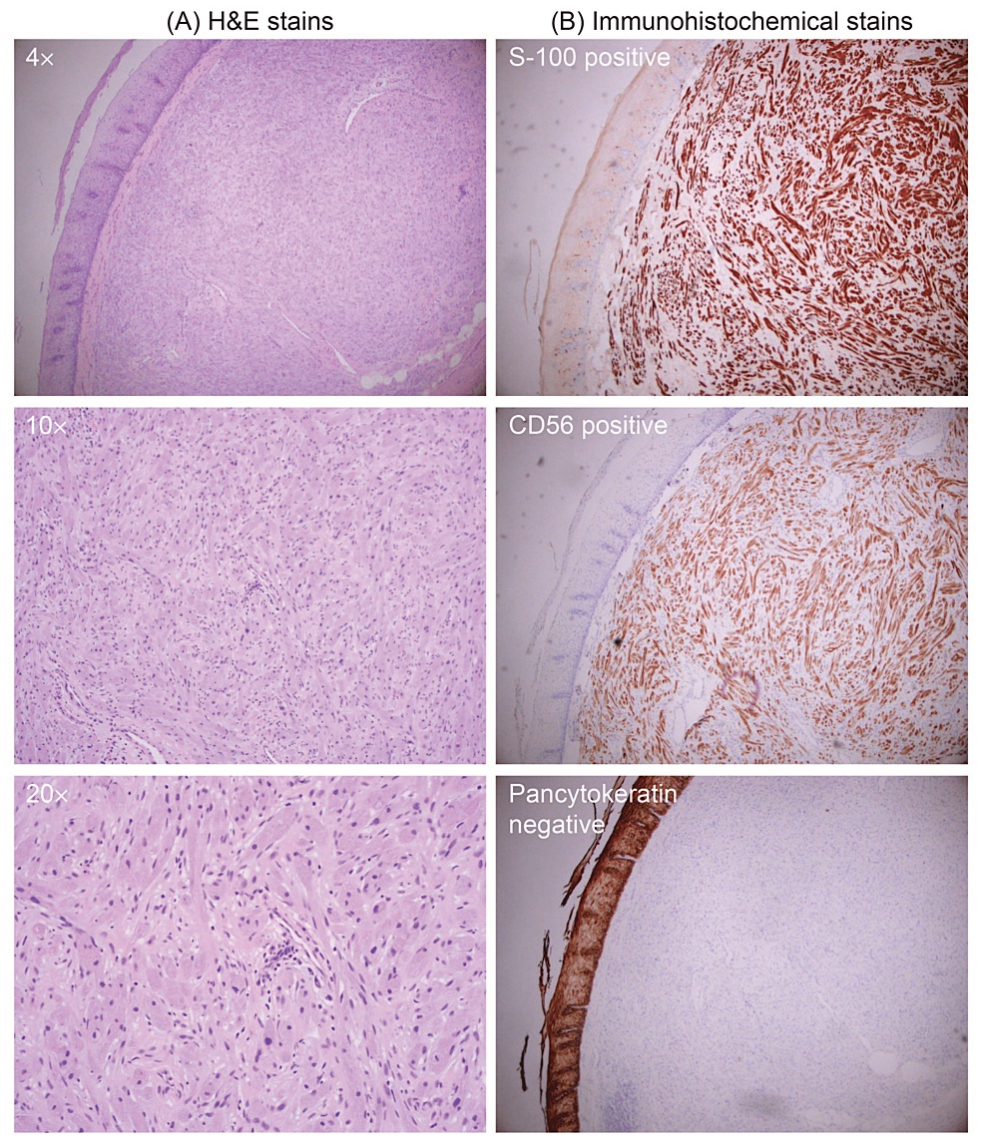
(A) Histologic evaluation of an H&E stained esophageal GCT showing nests and ribbons of oval-shaped cells with a central nucleus and abundant granular cytoplasm at magnifications of 4×, 10×, and 20×. (B) Immunohistochemical staining of the esophageal GCT shows that tumor cells are positive for S-100, positive for CD56, and negative for pancytokeratin. H&E, hematoxylin and eosin; GCT, granular cell tumor

Histopathological evaluation of a tissue biopsy stained with hematoxylin and eosin (H&E) revealed an unordered tangle of cellular ribbons intermixed with fibrous bands. Cytological features were marked by pleomorphic cell size and shape ranging from ovoid to spindle with cytoplasms dotted by plentiful eosinophilic granules, as seen in Figure 2A. Immunohistochemical staining confirmed the diagnosis of a GCT, with cells staining positive for S-100 and CD56 but negative for pancytokeratin, as shown in Figure 2B. 

The patient elected to have his esophageal GCT removed with endoscopic mucosal resection. A repeat EGD at nine months post-treatment was negative for new growth. 

## Discussion

First described in a tongue neoplasm by Abrikossoff in 1926, GCTs, also referred to as Abrikossoff tumors, are uncommon lesions known to arise almost anywhere in the body, including the oral mucosa, breast, vulva, skin, larynx, bronchus, biliary system, and esophagus [9,10]. Esophageal GCTs are very rare and are found in less than 0.02% of EGDs [5,7]. Initially, GCTs were thought to originate from muscle cells due to their granular appearance on histological examination; however, subsequent studies have provided histochemical data suggesting that GCTs arise from Schwann cells [9,11]. Irrespective of their location, they are usually asymptomatic and discovered incidentally. Patients presenting with a GCT have a median age near 50 years [2,3,5]. There is a slight female predominance, with a male-to-female ratio of 40:60 [2,3]. When GCTs arise in the gastrointestinal tract, they typically form in the distal esophagus, accounting for 65% of esophageal GCTs. In contrast, middle and proximal esophageal GCTs account for 20% and 15% of cases, respectively [5,12,13]. When symptomatic, the most common manifestation of an esophageal GCT is dysphagia, although acid reflux and abdominal pain may also be the chief complaint on initial presentation [5]. Esophageal GCTs causing dysphagia are more likely to be larger than 1 cm along their greatest dimension [14]. The patient discussed in this case report had an intramural esophageal GCT measuring 1.2 cm along its greatest dimension and presented with dysphagia.

Dysphagia describes the subjective sensation experienced by the patient whereby they find it difficult to swallow. This is distinct from odynophagia, which signifies pain with swallowing. Rapid onset dysphagia raises the suspicion of a foreign body lodged in the esophagus, with the most common etiology being food impaction. Conversely, when a patient complains of chronic dysphagia, a wide differential diagnosis must be narrowed with a focused history, physical exam, and diagnostic workup. An EGD can often rule out a structural abnormality, winnowing the differential diagnosis toward entities such as a motility disorder that should be investigated with esophageal manometry or gastroesophageal reflux disease. If a structural abnormality is present, the EGD may be able to stratify which type of mechanical aberration is present such as a stricture, web, ring, leiomyoma, hemangioma, gastrointestinal stromal tumor, rhabdomyoma, schwannoma, etc.

When esophageal subepithelial masses are discovered with EGD, it is essential to localize the origin of the lesion further to guide management. However, due to obfuscation by mucosa, EGD alone cannot distinguish between the possible subepithelial lesions nor even differentiate between an intramural versus extramural etiology [15]. The differential diagnosis for such a subepithelial esophageal mass is broad. It includes intramural tumors arising from within one of the four layers of the gastrointestinal wall (e.g., GCT, gastrointestinal stromal tumor, neuroendocrine tumor, lipoma, leiomyoma, and ectopic mass of pancreatic tissue) or extramural etiologies such as a large aneurysm of the aorta or splenic artery, pancreatic pseudocyst, or tumor of the left lobe of the liver.

EUS is typically the next step after EGD in the workup of a subepithelial mass of the esophagus [15-17]. EUS can guide diagnosis by providing information about the layer from which the lesion arises while also directing fine needle aspiration or fine needle biopsy. On EUS, GCTs conventionally appear as a solitary heterogeneous mass within the submucosa [15]. 

Analysis of tissue biopsies of GCTs is key to arriving at an ultimate diagnosis because they are associated with a characteristic pathological scheme. Staining of GCTs with H&E shows ill-defined masses consisting of large polygonal to round cells with abundant cytoplasmic granules [1]. The borders of these neoplasms are often irregular. Gross inspection will involve a small, firm, tan-yellow mass, typically about 1 cm along its greatest dimension, and a high likelihood of an ulcerated or eroded surface [1-3,18]. Immunohistochemical stains support a diagnosis of GCT. Given their Schwann cell origin, GCTs invariably stain positive for S-100 [2,19]. Other positive stains include CD68, CD56, SOX10, neuron-specific enolase, PGP9.5, and vimentin [19]. 

Although malignant behavior is rare for GCTs, their treatment mainly consists of removing the mass because of the underlying potential for malignancy and the danger of metastasis to vital organs that comes with malignant neoplasms [4]. Endoscopic mucosal or submucosal resection is typically employed; alternatively, surgical resection or cryoablation may be required in some instances [20]. The prognosis for patients with benign GCTs treated with tumor removal is excellent, with rare recurrence reports. Conversely, malignant GCTs are associated with a high chance for recurrence and a 5-year survival rate between 60% and 75%, with increasing tumor size or metastasis being two factors that worsen prognosis [4].

In our patient, a 68-year-old male with worsening dysphagia occurring mostly to solids but also to liquids, a single 12 mm intramural nodule was uncovered with EGD. Follow-up studies with EUS, mucosal biopsy, and histopathology revealed a mass of neoplastic cells containing eosinophilic granules, which stained positive for S-100 and CD56. All findings are consistent with a benign esophageal GCT. The subepithelial mass was successfully removed with endoscopic mucosal resection, and the patient has reported a total absence of dysphagia following treatment with no recurrence on repeat EGD. Two atypical features of this case are that (1) we present the case of a GCT in a male, whereas GCTs more commonly arise in women, and (2) this esophageal GCT was found in the middle third of the esophagus, but they typically form in the distal esophagus.

## Conclusions

GCTs are uncommon, typically benign neoplasms of Schwann cell origin most frequently found in the gastrointestinal tract, skin, and respiratory tract. Esophageal GCTs are rare, but they are normally found in the distal esophagus when they do arise. Given the broad differential diagnosis for dysphagia, EGD followed by EUS to determine lesion size, location, and to obtain image-guided tissue biopsies is needed to arrive at a final diagnosis. Pathological analysis is required for diagnosis where H&E staining will show nests of large round or oval cells with copious cytoplasmic granules. Immunohistochemical studies will aid diagnosis, as all GCTs will stain positive for S-100, CD68, CD56, SOX10, neuron-specific enolase, PGP9.5, and vimentin. The preferred treatment is removal via endoscopic mucosal resection or surgical resection. Although the potential for a malignant GCT is small, GCT removal is the recommended treatment in all cases so long as the patient is healthy enough to tolerate excision. Given the rare etiology but otherwise common presenting symptom of dysphagia as seen in our patient, GCT should not be missed as a differential in evaluating patients with difficulty swallowing.
